# Using the SOFA 2.0 score: a quick guide for clinicians and researchers

**DOI:** 10.62675/2965-2774.20250395

**Published:** 2025-11-13

**Authors:** Suzana Margareth Lobo, Roberta Muriel Longo Roepke, Sheila Nainan Myatra, Ederlon Rezende

**Affiliations:** 1 Hospital de Base Faculdade de Medicina de São José do Rio Preto Intensive Care Department São José do Rio Preto SP Brazil Intensive Care Department, Hospital de Base, Faculdade de Medicina de São José do Rio Preto - São José do Rio Preto (SP), Brazil.; 2 Universidade de São Paulo Faculdade de Medicina Department of Surgery Sâo Paulo SP Brazil Trauma and Acute Care Surgery Intensive Care Unit, Department of Surgery, Hospital das Clínicas, Faculdade de Medicina, Universidade de São Paulo - Sâo Paulo (SP), Brazil.; 3 Homi Bhabha National Institute Tata Memorial Hospital Department of Anaesthesiology Mumbai India Department of Anaesthesiology, Critical Care and Pain, Tata Memorial Hospital, Homi Bhabha National Institute - Mumbai, India.; 4 Instituto de Assistência Médica ao Servidor Público Estadual Hospital do Servidor Público Estadual de São Paulo Intensive Care Department Sâo Paulo SP Brazil Intensive Care Department, Hospital do Servidor Público Estadual de São Paulo, Instituto de Assistência Médica ao Servidor Público Estadual - Sâo Paulo (SP), Brazil.

## INTRODUCTION

Intensivists routinely assess organ dysfunction/failure using standardized tools. The original Sequential Organ Failure Assessment (SOFA) score^(1)^ was designed to describe, not predict, organ dysfunction, and although it has since demonstrated strong associations with morbidity and mortality in critically ill patients, it was not originally intended as a prognostic score. It assigns a 0 - 4 score across six organ systems, based on expert consensus and initially tested in small retrospective and prospective cohorts.^(2)^

SOFA has since been widely adopted in clinical practice and in research as the main organ dysfunction score. Scientific advancements in the field of intensive care medicine have supported the need for an updated version of the SOFA score,^(3-5)^ while current availability of large intensive care unit (ICU) registries and open access databases have provided a unique opportunity for development and data-driven validation of an updated score. However, substantial changes in ICU practices, the emergence of new organ support technologies, and the increasing need for equitable applicability across resource-rich and resource-constrained settings have created a demand for a modernized version. Furthermore, the growing integration of electronic health records (EHRs) and AI-driven decision support systems requires updated and digitally compatible scoring tools to facilitate automated calculation and real-time interpretation.

This viewpoint aims to highlight key changes in the newly released SOFA 2.0 and to provide guidance for its implementation in daily clinical practice.

### From original SOFA to SOFA 2.0 - key changes

The updated SOFA 2.0 score was developed through a rigorous methodological process, including a modified Delphi consensus among a diverse expert panel, systematic reviews, and final validation in over a million patients across multiple countries, healthcare systems, and geographic and economic regions. The original six organ systems were retained but updated to reflect current clinical practices by incorporating new definitions, additional variables, and revised thresholds to better categorize the severity of organ dysfunction, while preserving the score's core principles.^(6,7)^ This modernization ensures both clinical relevance and greater alignment with contemporary patterns of organ support and monitoring. Researchers have addressed key limitations of the original SOFA score, particularly in the respiratory, cardiovascular, and renal domains, to improve its ability to describe organ dysfunction and mortality risk across diverse ICU populations.^(5)^ Although the addition of new organ systems such as gastrointestinal and immune function was initially planned, these were ultimately excluded due to insufficient predictive and content validity, respectively.^(6,7)^

SOFA 2.0 retains the 0 - 4 grading system for each organ system, but the distribution of scores has shifted, particularly in the respiratory, cardiovascular, and renal systems. Respiratory system changes include the use of SpO_2_/FiO_2_ as an alternative to PaO_2_/FiO_2_, recognition of non-invasive respiratory support (e.g., high-flow nasal oxygen [HFNO], continuous positive airway pressure [CPAP]) as part of ventilatory support, and the inclusion of extracorporeal membrane oxygenation (ECMO). SOFA 2.0 also allows for scoring patients not receiving advanced respiratory support due to treatment limitations or lack of availability, using either PaO_2_/FiO_2_ or SpO_2_/FiO_2_ ratios. Cardiovascular system updates expand the range of vasopressors and inotropes, with equivalent dosing provided, and incorporate mechanical circulatory support into the scoring criteria. For the renal system, there is a shift in the previous cut-off values and inclusion of renal replacement therapy (RRT) or eligibility for RRT when unavailable. Novel changes for the brain include specific criteria outlined for sedated patients and, more importantly, the incorporation of delirium treatment as a marker of organ dysfunction. For the liver and coagulation systems, bilirubin levels and platelet counts remain the key parameters, but with updated thresholds and clearer operational definitions. Overall, these updates enhance consistency with contemporary clinical practice and broaden applicability across varying levels of resource availability.

**Figure 1:**
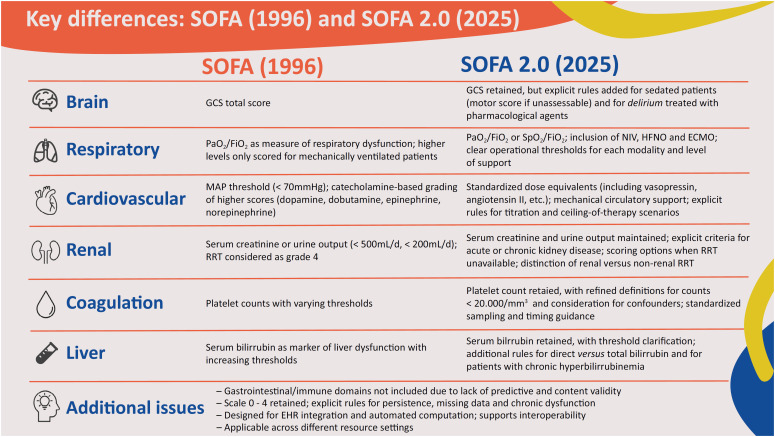
Key differences between the original SOFA and SOFA 2.0 scores.

SOFA - Sequential Organ Failure Assessment; GCS - Glasgow Coma Scale; NIV - noninvasive ventilation; HFNO - high-flow nasal oxygen; ECMO - extracorporeal membrane oxygenation; MAP - mean arterial pressure; RRT - renal replacement therapy; EHR - electronic health record.

The updates in SOFA 2.0 reflect contemporary clinical practices and aim to increase applicability across a range of ICU settings, including those with limited resources.^(6,7)^ By preserving the original score's conceptual simplicity while incorporating modern therapeutic modalities and broader accessibility criteria, SOFA 2.0 strengthens its clinical versatility and global usability.

Collectively, these revisions enable SOFA 2.0 to achieve what the original score could not fully support: a contemporary representation of organ dysfunction aligned with current ICU technologies, a more equitable scoring framework that accommodates settings with variable resource availability, and a structure compatible with automated digital integration. By refining definitions while maintaining its foundational simplicity, SOFA 2.0 strengthens its role as both a bedside assessment tool and a harmonized research instrument across diverse global contexts.

### Practical implications for clinical care

SOFA 2.0 was developed with detailed operational rules to enhance consistency and reproducibility in routine clinical practice. Building on the original SOFA concept of sequential assessment, the updated version provides explicit guidance on how to interpret and score values according to their persistence and clinical significance, ensuring uniformity across observers. Clear instructions are included for patients with chronic organ dysfunction (e.g., RRT or long-term mechanical ventilation), distinguishing baseline abnormalities from acute deterioration. The new framework also defines standardized approaches for handling missing data and for cases where a ceiling of treatment precludes advanced organ support, allowing appropriate surrogate variables.^(8)^

SOFA 2.0 facilitates integration into EHRs and automated data systems, enabling real-time calculation, reducing human error, and supporting continuous monitoring of organ dysfunction both at the bedside and across multicenter registries.^(6,7)^ User-oriented resources, including an operational manual and calculator, are forthcoming to assist with consistent application. This digital compatibility supports more timely clinical decision-making and enhances consistency in patient monitoring across teams and institutions.

### Implications for research

The transition to SOFA 2.0 will affect research frameworks that rely on the original score for sepsis definitions (Sepsis-3),^(9)^ trial eligibility, and benchmarking. Updated variables, such as SpO_2_/FiO_2_, HFNO, noninvasive ventilation, vasopressin, angiotensin II, and ECMO, reflect contemporary practice but may challenge established datasets and risk models. To preserve comparability and data continuity, implementation should follow a phased dual-scoring approach, allowing calibration between original SOFA and SOFA 2.0 while supporting the generation of validated crosswalk mappings. This strategy safeguards ongoing studies and registries while enabling harmonization across centers and income settings.

By aligning research definitions with contemporary clinical realities, SOFA 2.0 creates a common language that not only enhances scientific rigor but also facilitates its translation into bedside decision-making. In this way, harmonized scoring supports a continuous feedback loop between research and practice, where evidence guides implementation and clinical experience refines future investigations.

Future research should focus on trajectory-based endpoints and the integration of frailty and comorbidity indices to enhance calibration and clinical relevance in heterogeneous ICU populations. Additionally, longitudinal validation in diverse epidemiological contexts will be critical for assessing the prognostic robustness and adaptability of SOFA 2.0.

### Guidance for implementation

The SOFA 2.0 score provides a structured framework that can be seamlessly incorporated into both bedside assessment and research workflows. For clinicians, its standardized definitions and explicit scoring rules allow for reliable daily evaluation of organ dysfunction, even across teams with varying levels of experience. Consistent use promotes better communication among care providers and supports clinical decisions based on objective, comparable trends rather than subjective impressions. For researchers, the score's harmonized variables and thresholds facilitate reproducible data collection and uniform interpretation across studies, enabling more accurate comparisons between cohorts and interventions.

Implementation should include team-training programs emphasizing practical case-based exercises, integration into EHR with automated data capture, and institutional protocols that embed SOFA 2.0 into daily rounds and quality monitoring. To further support real-world adoption, user-oriented materials such as an operational manual and digital calculators are under development to facilitate consistent and efficient application in both clinical and research settings.

### Limitations and future perspectives

Although SOFA 2.0 was developed using extensive international datasets from heterogeneous ICU populations, it still requires prospective validation to confirm reproducibility in real-world workflows across varied healthcare systems and disease profiles.^(10)^

The exclusion of gastrointestinal and immune dysfunction, despite being frequently evaluated by intensivists, reflects current limitations in available datasets and operational feasibility at the bedside. Additionally, the score must be tested in resource-constrained environments and high-burden disease regions (e.g., malaria, HIV, dengue) to ensure equitable application. Cultural variability in end-of-life decision-making may also affect observed outcomes and model calibration, highlighting the need for contextualized validation and careful interpretation.

SOFA 2.0 is not intended as a static endpoint but rather as a foundation for a dynamic, data-informed framework for organ dysfunction assessment. As it becomes integrated into EHRs, automated scoring and trajectory-based monitoring will support real-time clinical decision-making and enable linkage to predictive analytics and AI-driven decision support systems. Future iterations should incorporate emerging technologies, enhance adaptability in low-resource settings, and explore integration of complementary constructs such as frailty, comorbidities, subphenotypes, and biomarkers. Sustained global adoption will require iterative re-evaluation informed by performance monitoring, evolving evidence, and international feedback.

The next steps for the SOFA 2.0 community should prioritize:

– Conducting robust prospective validation in heterogeneous clinical environments, including resource-limited settings and regions with high prevalence of emerging and neglected diseases.
– Developing and deploying digitally integrated tools, including automated EHR-based scoring, trajectory monitoring, and decision-support algorithms to enhance real-time clinical utility.
– A digital architecture also opens the pathway for progressive integration with artificial intelligence and big data analytics, enabling living updating of the score in response to evolving evidence and the future trajectory of critical care.

## CONCLUSION

SOFA 2.0 preserves the conceptual simplicity and descriptive purpose of the original score while aligning organ dysfunction assessment with contemporary intensive care practices. Its standardized definitions enhance clinical and research consistency, promote interprofessional communication, and enable reliable monitoring across diverse care settings. The updated structure supports equitable application in both high- and low-resource environments and facilitates integration into digital health ecosystems.

As critical care continues to evolve in a data-driven and globally connected landscape, SOFA 2.0 provides a foundation for real-time monitoring, predictive analytics, and harmonized benchmarking. Rather than a static endpoint, it represents a pivotal step toward a continuously adaptive model of organ dysfunction assessment informed by iterative evaluation and technological innovation.
